# Colonic Gastrointestinal Stromal Tumor: A Population-Based Analysis of Incidence and Survival

**DOI:** 10.1155/2019/3849850

**Published:** 2019-04-11

**Authors:** Zhiqiang Liu, Yan Sun, Yongfeng Li, Jingyuan Zhao, Shihong Wu, Zibo Meng, Heshui Wu

**Affiliations:** ^1^Department of Pancreatic Surgery, Union Hospital, Tongji Medical College, Huazhong University of Science and Technology, Wuhan 430022, China; ^2^Department of Gastrointestinal Surgery, Union Hospital, Tongji Medical College, Huazhong University of Science and Technology, Wuhan 430022, China

## Abstract

**Objectives:**

The incidence of gastrointestinal stromal tumors (GISTs) located in the colon is rare. Current studies mainly focus on case reports for colonic GISTs. Therefore, a population-based analysis was useful to guide the clinical treatment strategy.

**Methods:**

The patients were selected from 2000 to 2015 based on Surveillance, Epidemiology, and End Results (SEER) database. Patients' demographics, tumor characteristics, incidence, treatment, and survival were retrieved for analysis.

**Results:**

249 cases of colonic GISTs were collected. The male-female ratio was close to 1 : 1 (male 51.41%, female 48.59%). Most cases were Caucasians (70.28%), and African Americans accounted for 19.68%. Age of diagnosis ranged from 21 to 93 years with a median (mean) age of 67.5 (65.56). The incidence was rare, only 0.018 per 100,000. It had an annual percentage change (APC = −0.7728) without statistical significance (*P* = 0.5127) while the incidence of other GISTs increased from 2000 to 2015, with an annual percentage change of 3.9% (*P* = 0.0001). Surgery was associated with better prognosis whereas chemotherapy did not impact the survival rate.

**Conclusion:**

Colonic GIST is a rare solid tumor, and the incidence is stable. The entity has a poorer prognosis than other GISTs. Surgery improved the survival rate, while chemotherapy did not.

## 1. Introduction

Gastrointestinal stromal tumors (GISTs), caused by interstitial cells of Cajal, have been proven to be the most common mesenchymoma in the gastrointestinal tract [1]. It is reported that the morbidity rate of GISTs is between 11 and 15 people per million [2]; however, an increasing number of studies have found out that it disappreciates seriously [3, 4].

GISTs mainly occur in the digestive tract, such as the stomach (70%), small intestine (20%-30%), and colon and rectum (10%) [5]. Although some inspiring progress has been made about the treatments of GISTs, surgical resection is still the main treatment method. The 5-year survival rate is 48-70% after a local GIST resection, and the recurrence rate is 40-80% even though a histopathologically complete resection of the tumor is done [6]. When the tumor recurs or metastasizes, imatinib, a small-molecule tyrosine kinase inhibitor [7], is used to kill tumor cells to improve prognosis [8].

It is challenging to estimate the invasiveness and malignant potential of GISTs. There are various influencing factors in the prognosis of GISTs, including tumor size, mitotic rate, tumor ulceration, sex, age, symptoms, and IHC results [9, 10]. Besides, the tumor location is also considered a predictive index, and nongastric disease location can lead to a worse prognosis [2]. However, studies on colonic GISTs are rare at present, and the literature is mainly case reports or small case series. Therefore, we utilized the population-based SEER database to investigate the incidence and survival trend of colonic GISTs, as well as to elucidate epidemiologic traits, treatment modalities, staging frequency, and survival outcome, thus providing new insight for colonic GISTs.

## 2. Materials and Methods

### 2.1. Patient and Tumor Characteristics

Some basic information, including frequency, incidence, and survival data, for colonic and other GISTs between 2000 and 2015 were extracted from SEER. Because patient identifiers were omitted in the SEER 18 database, this research finds it unnecessary to get the approval of the Institutional Review Board (IRB).

The SEER 18 database was selected utilizing a histology/behavior code (8936) corresponding to GISTs based on International Classification of Diseases for Oncology, 3rd Edition (ICD-O-3). We divided the search results into two parts: one group is called colonic GISTs as the tumors are located in the colon and the other is called other GISTs as tumors are located elsewhere in the digestive tract. We conducted classification and statistics for the weight of patient characteristics in the light of sex, ethnicity, marital status, age, tumor location, and grade. We classified tumor characteristics by utilizing the tumor-node-metastasis (TNM) staging and the American Joint Committee on Cancer (AJCC) staging and operation method. Patients who survived to the deadline and patients who were not followed up or died of other reasons belonged to the right-censored data for the overall survival (OS) and cancer-specific survival (CSS) analysis.

### 2.2. Incidence and Survival

All rates were reported per 100,000 persons, and age of the cases was adapted for the 2000 US Standard Population (19 age groups, census P25-1130) standard. The annual percentage change (APC) was also figured out in incidence using 1-year endpoints to analyze the survival rate. Two indicators were used to analyze the survival trends—OS and CSS. Results of the OS and CSS by surgery modality and tumor staging were also obtained through Kaplan-Meier analysis.

### 2.3. Statistical Analysis

Frequency, incidence, and survival data of all cases were extracted and analyzed from the SEER 18 database by utilizing the SPSS software (version 20.0, SPSS Inc., Chicago, IL, USA), MedCalc statistical software (version 15.2.2, MedCalc Software bvba, Ostend, Belgium), or SEER∗Stat 8.3.5 software (National Cancer Institute, Bethesda, Maryland). The incidence data were analyzed through weighted least squares to generate annual percentage change based on 1-year endpoints using the SEER∗Stat 8.3.5 software. Student's *t*-test was used to compare continuous variables, whereas as for categorical variables, we adopted the chi-square test for analysis. A *P* value of <0.05 indicates statistical significance, and all *P* values were two-tailed.

## 3. Results

### 3.1. Patient Characteristics

**Figure 1:**
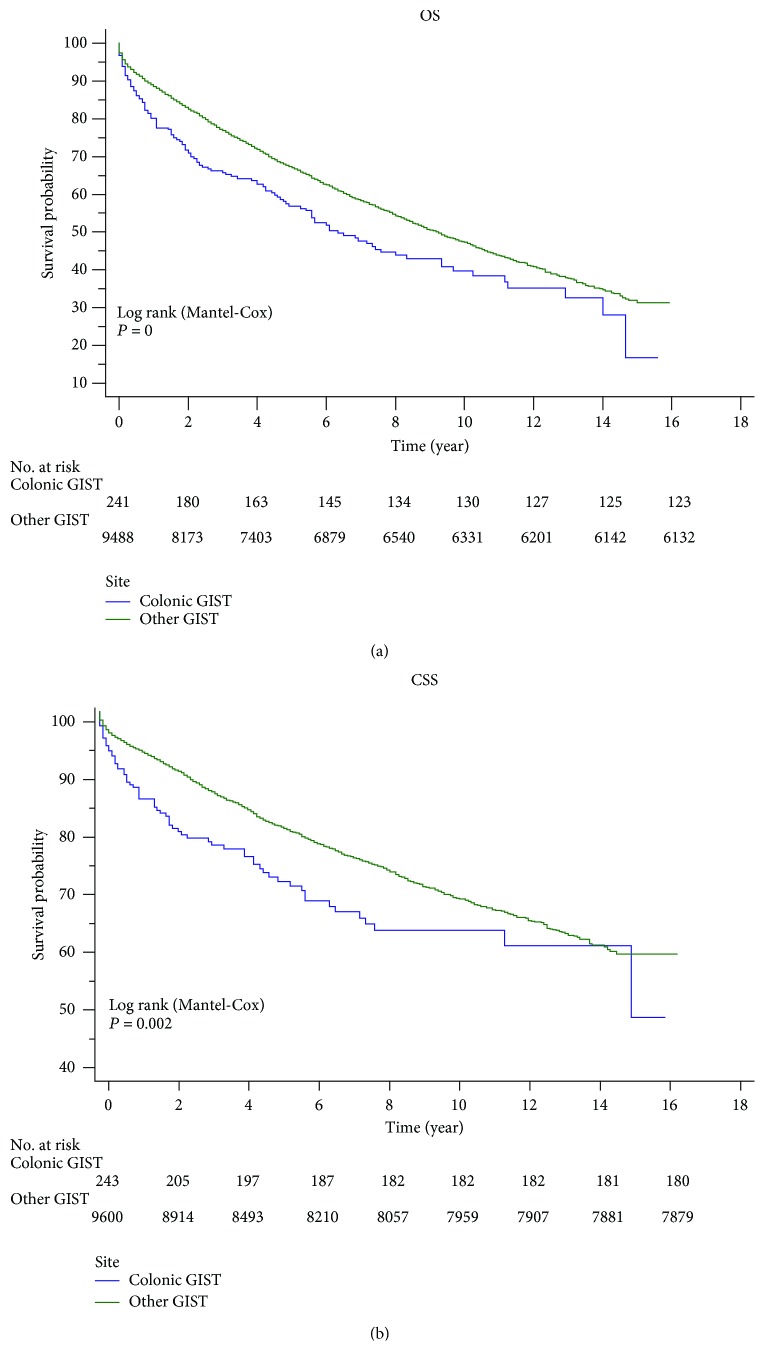
Survival analysis between colonic GISTs and other GISTs in OS/CSS.

A total of 10046 GIST patients were collected in which 56 cases with an unknown tumor site were excluded. 249 colonic GIST and 9741 other GIST cases were included. The demographic characteristics are demonstrated in Table 1. Colonic GISTs were found to be more common in males with a slight male predominance (male 51.41%, female 48.59%) which was consistent with previous studies [11–13]. 55.82% of the patients were married, and most cases were Caucasians (70.28%), while African Americans accounted for 19.68% and others represented 10.04%. The distribution of the corresponding proportions in other GISTs is similar to that in colonic GISTs (*P* = 0.706, *P* = 0.370, and *P* = 0.342, respectively). The age of onset ranged from 21 to 93 with a median (mean) age of 67.5 (65.56) in colonic GISTs whereas in other GISTs, the age range was from 8 to 101 and the median (mean) was 64 (63.17) (*P* = 0.029). Besides, more colonic GISTs were in higher-grade lesions than other GISTs (20.08% vs. 9.76%, *P* = 0). According to the ICD-O-3 primary site code, most (57.76% and 27.20%) cases of other GISTs were located in the stomach and the small intestine, whereas about 11.162%cases did not arise in the digestive tract (Table 1). Similar to previous reports [11, 14], GISTs located in the colon demonstrated poorer OS and CSS than other GISTs (Figure 1). However, the survival time showed no remarkable difference between the two groups (Table 1).

### 3.2. Tumor Characteristics

Tumor-node-metastasis (TNM) staging and American Joint Committee on Cancer (AJCC) staging are summarized in Table 2. We used the AJCC seventh edition staging system for there is no foreseeable change in the AJCC eighth edition for GIST [15]. In colonic GISTs, T1 tumors were the most commonly identified (30.53%), followed by a lesser proportion of T2 (18.95%), T3 (15.79%), and T4 (12.63%) (*P* = 0). Lymph node involvement was infrequent with N0 of 91.58% (*P* = 0). As for distant metastasis, metastasis (M1) occurred in 15.79% of cases and 78.95% did not have distant metastasis. The AJCC staging was arranged according to the proportion from high to low in colonic GISTs—I (27.37%), III (18.95%), IV (15.79%), and II (6.32%) (*P* = 0.001).

### 3.3. Incidence Analysis

**Figure 2:**
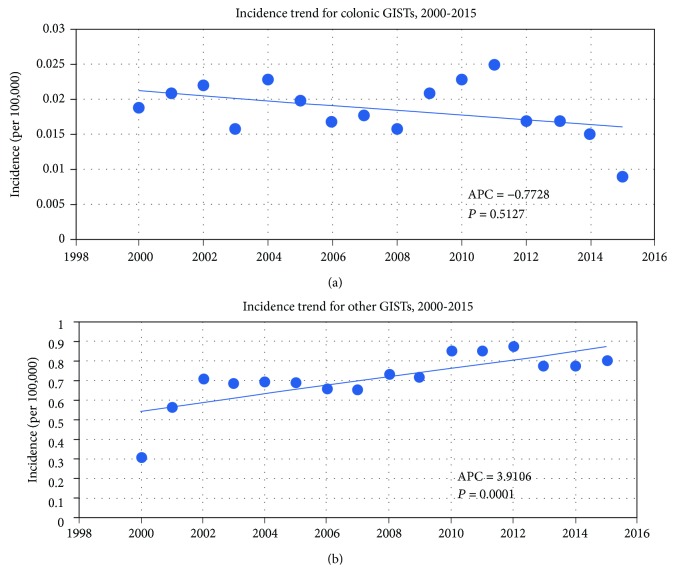
Incidence trend for colonic GISTs and other GISTs.

The incidence of colonic GISTs was rare, which was ascertained to be 0.018 per 100,000 between the years 2000 and 2015 based on age adjusted to the 2000 US Standard Population (19 age groups, census P25-1130) standard (Table 1). Although the annual percentage change (2000-2015) was -0.7728%, the slight decrease of incidence lacked statistical significance (*P* = 0.5127). However, the incidence of other GISTs was 0.719 per 100,000, with an annual percentage change (2000-2015) of 3.9106% (*P* = 0.0001) (Table 1, Figure 2).

### 3.4. Treatment and Survival Analysis

The trends in therapy are summarized in Table 3. Surgery was performed in 79.52% of colonic GISTs and 77.35% of other GISTs, which presents a statistically significant tendency (*P* = 0). In colonic GIST cases, the largest proportion of surgery was total excision (35.34%) and partial excision on its heels (31.33%), while partial excision was the most common (44.45%) in other GISTs. Chemotherapy was used in 31.33% of colonic GISTs, compared with 39.13% for other GISTs, showing a statistically significant difference (*P* = 0.013).

**Figure 3:**
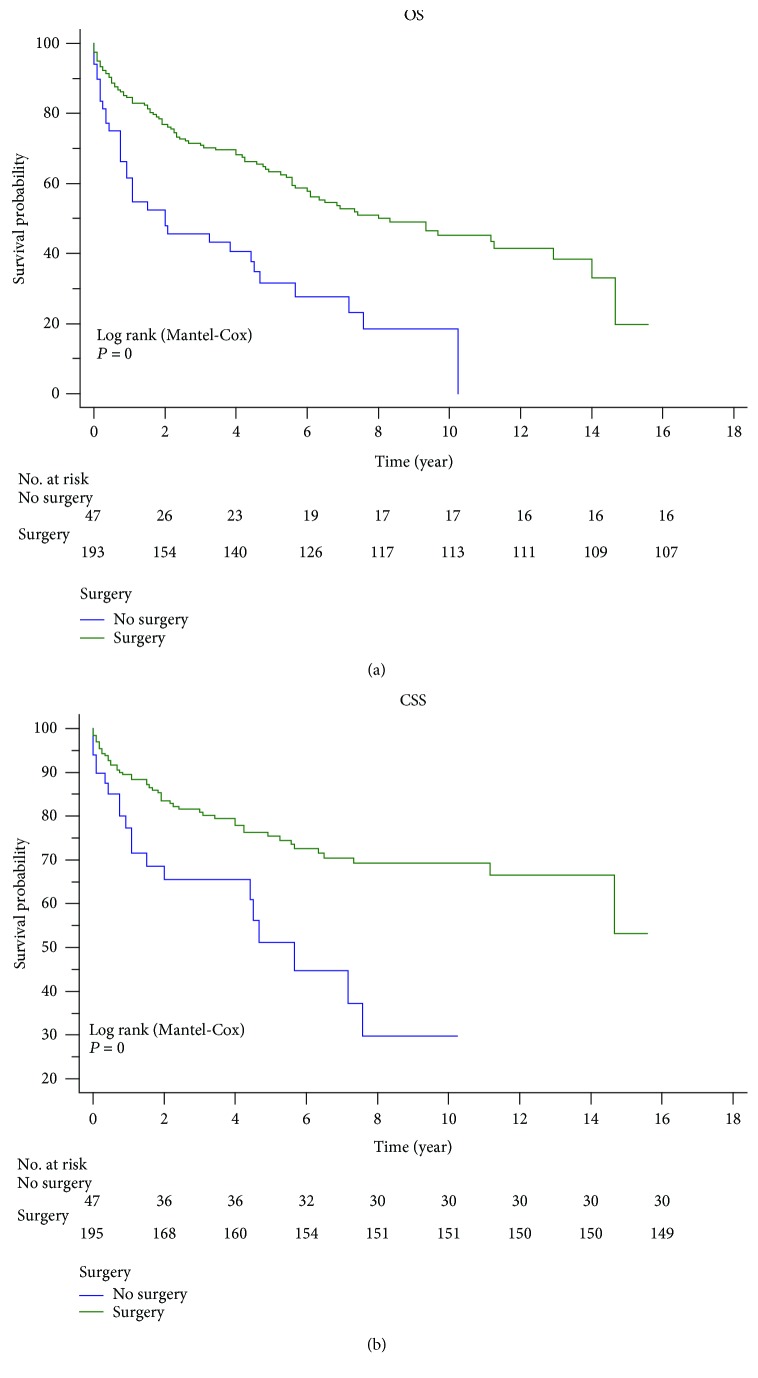
Surgery significantly improved the OS and CSS rates via Kaplan-Meier analysis of colonic GISTs.

**Figure 4:**
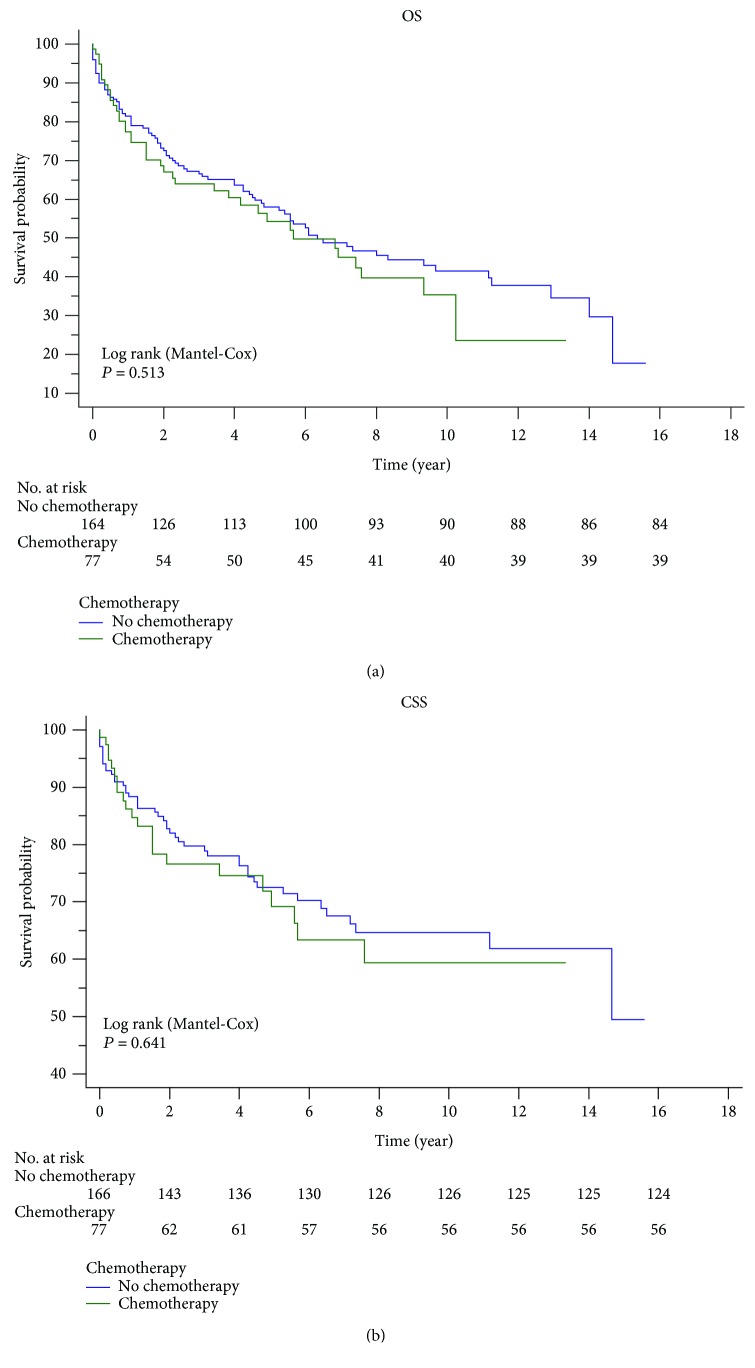
Chemotherapy did not improve the OS and CSS rates via Kaplan-Meier analysis of colonic GISTs.

**Figure 5:**
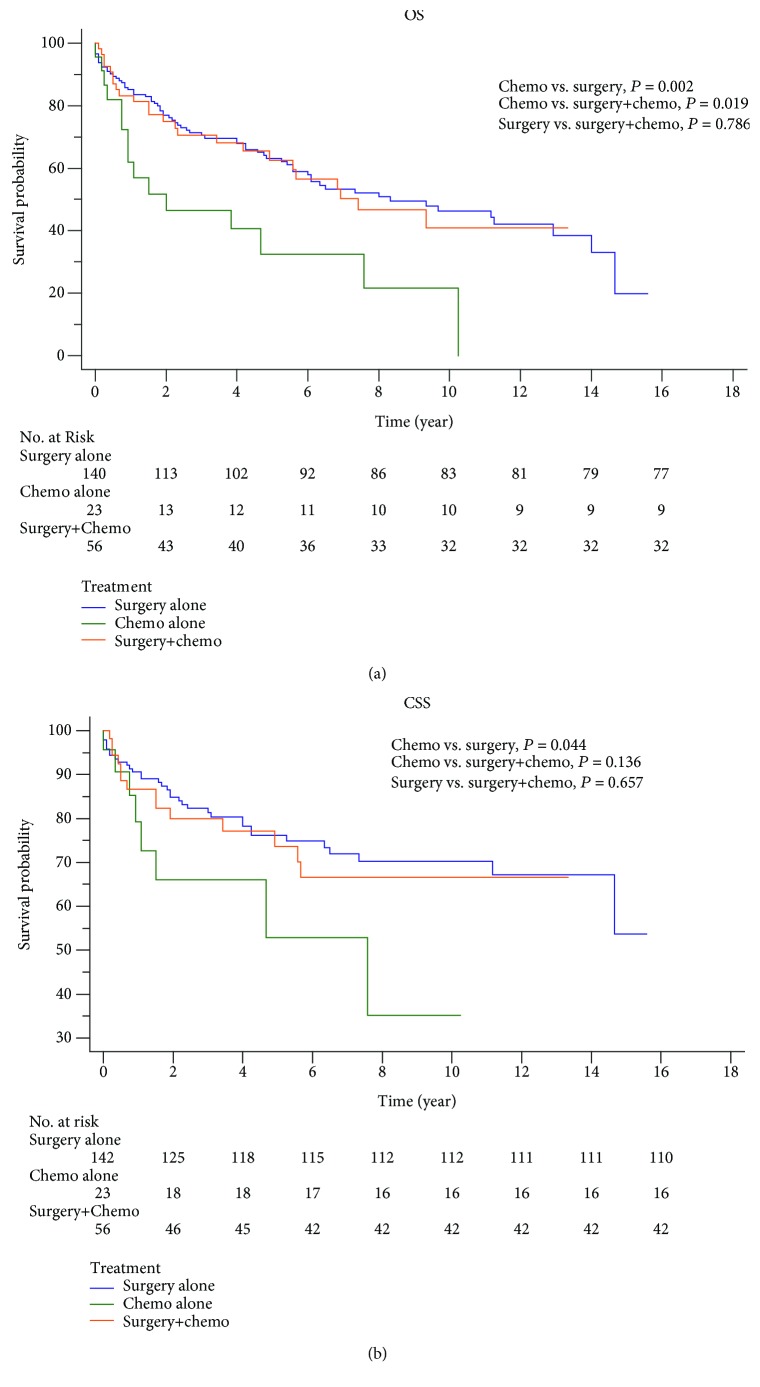
Kaplan-Meier analysis between different therapy modalities in colonic GISTs.

Colonic GISTs showed poorer prognosis than other GISTs (Figure 1). The survival rate was used to evaluate the effectiveness of surgery and chemotherapy for cancer treatment. Surgery significantly improved the OS and CSS rates via Kaplan-Meier analysis for colonic GISTs (Figure 3). However, chemotherapy did not have an advantageous effect to improve the five-year OS (no chemotherapy: 57.8%, chemotherapy: 54.8%, *P* = 0.686) or CSS (no chemotherapy: 72.3%, chemotherapy: 69.6%, *P* = 0.705) (Figure 4). Besides, we also performed a comparison among the three treatment modalities, including surgery alone, chemotherapy alone, and combined therapy (Figure 5).

## 4. Discussion

Approximately 75-85% of GIST arises in the stomach and the small intestine [16]. Colonic GIST reportedly constitutes about 2.9-9.3% of GIST [14, 17, 18]. Studies on colonic GIST are limited by its rarity. Most literature describing the characteristics, incidence, survival, and treatment strategies of colonic GIST are small case series or case reports [19–23]. The rarity of colonic GIST was confirmed by our analysis, presenting an incidence of 0.018 per 100,000 between 2000 and 2015 (Table 1, Figure 2) and accounting for 2.49% of GISTs (Table 1). However, APC in the incidence of colonic GIST does not significantly change between 2000 and 2015. Meanwhile, other GISTs demonstrate a notable APC increase (APC 3.9106%, *P* = 0.0001) (Table 1, Figure 2). Due to the inconsistencies of incidence between colonic and other GISTs, we sought to do a population-based large sample size analysis in an effort to elucidate the baseline characteristics, prognosis, and treatment modalities of this malignancy. We revealed a very interesting trend in colonic GIST using the SEER database.

Male sex, the age of diagnosis, and marital status were reported to be independent risk factors for GISTs [11, 13, 24]. Colonic and other GIST groups were similar in terms of sex, age, and marital status distribution (Table 1). Patients in the colonic GIST group presented with a higher percentage of high-grade lesions, are diagnosed at an older age, and had poorer prognosis (Tables 1 and 2 and Figure 1). About half of colonic GISTs were in the early T stage (T1, 30.53%; T2, 18.95%; Table 2) while most colonic GISTs (N0, 91.58%; M0, 78.95%) had no lymph node involvement and distant metastasis (Table 2). The percentage of GIST in AJCC stage I/II was almost equal to that in AJCC stage III/IV (Table 2).

Our analysis validated previous studies regarding the decreased survival of colonic GISTs compared with other GISTs [14, 17]. We demonstrated that OS and CSS were significantly higher in other GISTs than in colonic GISTs (Figure 1). 5-year CSS of 71.5% in our study is higher than the reported 61.5% in prior studies for colonic GISTs [17], but with similar 5-year OS. The descended prognosis of colonic GISTs compared with other GISTs demonstrated the differences in the behavior of this malignancy.

Surgery is a potentially curative treatment for primary GISTs and is the gold standard of resectable GISTs [25]. In our cohort, about four-fifths of patients with colonic GIST underwent surgery, and patients who underwent surgery showed a significantly higher OS and CSS compared with those who did not (Figure 3). The largest proportion of excision was total excision (35.34%) and partial excision on its heels (31.33%), whereas partial excision was the most common (44.45%) in other GIST patients (Table 3). The reason might be discriminating the degree of invasion nature between colonic and other GISTs [14].

In 2002, the first tyrosine kinase inhibitor (TKI), also called imatinib, was approved by the United States Food and Drug Administration (FDA) for the treatment of metastatic or unresected GISTs for the impressive effect [26]. The following studies confirmed its effects on neoadjuvant therapy and long-term therapy [18, 27]. Unfortunately, in our study, we were unable to analyze the effect of TKIs, since the SEER database does not contain specific information for chemotherapy. The K-M analysis reflected that chemotherapy illustrated no significant improvement of OS and CSS compared with the group without chemotherapy (Figure 4). When it came to the comparison of patients by therapeutic modalities, some interesting trends were noted (Figure 5). Patients in the group that only had surgical treatment had a significantly greater OS/CSS than those in the group who only had chemotherapy and who showed around 30/20 percent of increased OS/CSS (Figure 5). The group who only had surgical treatment had very similar survival rates compared with the group who had surgical treatment and chemotherapy (Figure 5). Interestingly, survival analysis found that patients who undertook surgery and chemotherapy had improved OS compared with the patients who undertook chemotherapy alone (*P* = 0.019, Figure 5), but not CSS (*P* = 0.136, Figure 5). The reason for this phenomenon might be the small number of people in the group who have undergone chemotherapy alone (*n* = 23, Figure 5). The other speculative reason might be that the patients who undertook chemotherapy alone or surgery plus chemotherapy treatment was in more advanced AJCC stages where surgery was not feasible. Unfortunately, we were unable to analyze this due to massive missing data of the AJCC stage (61.85%).

Despite the SEER database providing a nationally representative insight into rare colonic GIST, there were certain limitations. The recurrence of GISTs was not rare as what studies revealed in the past [25, 28, 29]. However, SEER does not contain sufficient information about recurrence. Some factors such as resection margin and use of chemotherapy [25] which affected survival rates were also not provided in SEER. Additionally, staging information was available for only 65 colonic GIST cases; the low number of cases might affect our conclusion due to selection bias. Even with these considerations, this study contributes to an analysis of the largest sample size of colonic GIST to date. We describe the behavior of the infrequent colonic GIST via the population-based resource and provide a better understanding of baseline characteristics, incidence, management strategies, and prognostic outcome.

## 5. Conclusion

A colonic gastrointestinal stromal tumor is a rare solid tumor with a very low and basically stable incidence. About half of the tumors are in the early T stage. The probability of lymph node involvement and distant metastasis is exceedingly rare. Surgery resection remains the primary choice to improve survival.

## Figures and Tables

**Table 1:** Patient demographics and incidence.

		Colonic GIST (*N* = 249)	Other GISTS (*N* = 9741)	*P*
Sex, *n* (%)	Male	128 (51.41)	5125 (52.61)	0.706
	Female	121 (48.59)	4616 (47.39)	

Race/ethnicity	White	175 (70.28)	6694 (68.72)	0.370
	Black	49 (19.68)	1741 (17.87)	
	Other	25 (10.04)	1269 (13.03)	
	NA	0	37 (0.38)	

Marital status	Married	139 (55.82)	5567 (57.15)	0.342
	Other	92 (36.95)	3673 (37.71)	
	NA	18 (7.23)	501 (5.14)	

Age	Range	21-93	8-101	0.029
	Median	67.5	64	
	Mean	65.56	63.17	

Grade	Grade I	19 (7.63)	1092 (11.21)	0.000
	Grade II	18 (7.23)	909 (9.33)	
	Grade III/IV	50 (20.08)	951 (9.76)	
	NA	162 (65.06)	6789 (69.70)	

Survival months	Range	0-187	0-191	0.470
	Median	43	44	
	Mean	54.78	55.19	

Location	Colon	249	NA	
	Stomach	NA	5626 (57.76)	
	Small intestine	NA	2650 (27.20)	
	Rectum and anus	NA	276 (2.83)	
	Esophagus	NA	56 (0.57)	
	Other	NA	1132 (11.62)	

Incidence		0.018	0.719	

Annual percentage change (2000-2015)		-0.7728 (*P* = 0.5127)	3.9106 (*P* = 0.0001)	

Rates are per 100,000, and age is adjusted to the 2000 US Standard Population (19 age groups, census P25-1130) standard.

**(a):** 

TNM	Colon			
		*n*	%	*P*
T	T0	0	0	0
	T1	29	30.53	
	T2	18	18.95	
	T3	15	15.79	
	T4	12	12.63	
	Tx	16	16.84	
	NA	5	5.26	

N	N0	87	91.58	0
	N1	3	3.16	
	Nx	5	5.26	

M	M0	75	78.95	0
	M1	15	15.79	
	Mx	5	5.26	

**(b):** 

Colon			
AJCC	*n*	%	*P*
I	26	27.37	0.001
II	6	6.32	
III	18	18.95	
IV	15	15.79	
NA	30	31.58	

**Table 3:** Therapy for colonic GISTs and other GISTs.

	Colon (%)	Other sites (%)	*P*
Surgery type			0
No surgery	50 (20.08)	2117 (21.73)	
Local excision	11 (4.42)	1144 (11.74)	
Partial excision	78 (31.32)	4330 (44.45)	
Total excision	88 (35.34)	532 (5.46)	
En bloc	14 (5.62)	1324 (13.59)	
Surgery NOS	7 (2.81)	205 (2.10)	
Unknown	1 (0.4)	89 (0.91)	
Chemotherapy			0.013
Chemotherapy	78 (31.33)	3812 (39.13)	
No chemotherapy	171 (68.67)	5929 (60.87)	

## Data Availability

The data analyzed during the current study are available in SEER data (1973–2015) (https://seer.cancer.gov/data/).
